# Real-time quantification of pollen release: from liquid suspensions to airborne counting

**DOI:** 10.1093/jxb/erag153

**Published:** 2026-05-20

**Authors:** Stephen L Buchmann

**Affiliations:** Department of Entomology, and Department of Ecology and Evolutionary Biology, The University of Arizona, Tucson, AZ, USA

**Keywords:** Aerobiology, buzz pollination, pollen, pollen counting, wind pollination

## Abstract

This article comments on:

**Lazarus BS, Wieser VC, Fieber B, Dellinger AS.** 2026. Pollen counting made easy: mobile pollen counter provides real-time results in the field. Journal of Experimental Botany **77**, 2901–2913. https://doi.org/10.1093/jxb/erag047

This article comments on:

**Lazarus BS, Wieser VC, Fieber B, Dellinger AS.** 2026. Pollen counting made easy: mobile pollen counter provides real-time results in the field. Journal of Experimental Botany **77**, 2901–2913. https://doi.org/10.1093/jxb/erag047

**Quantifying pollen amounts released from anthers after dehiscence remains one of the most labor-intensive and methodologically constraining tasks in pollination biology. Accurate pollen counts underpin diverse lines of inquiry, including the study of buzz-pollinated flowers, wind-pollinated species, pollen-dispensing schedules, and the biomechanics of pollen ejection. Yet, despite the centrality of pollen quantification to plant reproductive ecology, many commonly used methods remain slow, destructive, and prone to multiple sources of error (Ruedenauer *et al*., 2016; Minnaar and Anderson, 2019; Vallejo-Marín, 2022). These limitations have constrained comparative studies and impeded efforts to measure pollen release dynamically, particularly under realistic mechanical or aerodynamic conditions. Against this backdrop, the Technical Innovation paper by Lazarus *et al.* (2026) introduces a notable methodological advance: the use of a handheld airborne particle counter to quantify pollen in real time, either in the field or in the laboratory, directly from the air during buzz- or wind-mediated pollen release. By shifting pollen quantification from liquid suspensions to airborne sampling, the authors propose a fundamentally different way of conceptualizing pollen as a measurable biological signal.**

## Historical approaches and their persistent limitations

Few areas in plant reproductive biology have been so persistently constrained by methodological inertia as the quantification of pollen grains from individual anthers or entire flowers (Harder *et al*., 2016; Minnaar and Anderson, 2019). Classical approaches relied on softening anthers with lactic acid, staining pollen (commonly with acetocarmine), mounting pollen on slides, and manually counting typically 500–700 grains under ×400 magnification. Although time-consuming, these preparations could be recounted by the same or another person to check for accuracy, or multiple counts could be averaged and reported. Large anthers were often cut into halves or quarters and prepared on multiple slides. Overlapping of pollen grains was often a problem, resulting in inaccuracies in pollen counts.

Later, photographic methods and automated counting were developed (e.g. using the software ImageJ from NIH). One improvement in pollen counting adapted biomedical technology in the form of a hemacytometer, a blood cell-counting device. In this approach, pollen grains from an anther or flower were suspended in water, often with sonication to promote dispersion into monads, and then a known aliquot (typically 10 μl) of the pollen grain suspension was loaded onto the hemacytometer counting grid under an optical microscope. Pollen was still counted manually, followed by application of a formula to estimate the true number in the original floral volume. In general, this was a faster and more reliable method of counting than simple slide counts alone. When carefully executed, such methods could provide accurate estimates, but they remained time-consuming and subject to coincidence errors, especially in species producing large pollen loads or aggregated grains (Ruedenauer *et al*., 2016).

More recent protocols have incorporated image-assisted counting and automated pipelines adapted from biomedical particle analysis software (Seipel *et al*., 2021). While these developments reduced observer bias and manual effort, they did not eliminate the need for extensive sample preparation, taxon-specific calibration, or destructive processing of pollen samples. A common feature of these approaches is their retrospective nature: pollen is collected, processed, and counted only after release has occurred. As a result, real-time dynamics of pollen ejection—central to understanding buzz pollination, pollen dosing, and dispersal trajectories—remain difficult to quantify directly.

## Liquid particle counters and the problem of pollen clumping

Liquid particle counters, including laser-based and Coulter-style devices, are widely regarded as a modern alternative for pollen quantification (Ruedenauer *et al*., 2016; Kakui *et al*., 2020). These instruments rapidly measure size-resolved particle distributions in suspension and can process large numbers of samples with minimal observer intervention. However, their performance depends critically on careful sample preparation and calibration.

A pervasive challenge is pollen clumping. Hydration of pollen in aqueous solutions can induce swelling, partial dissolution of pollenkitt lipids, or sporodermal adhesion, depending on the taxon (Dellinger *et al*., 2019; Pacini and Franchi, 2020). In species with sculptured exines or lipid-rich pollen coats, grains may adhere strongly upon suspension, forming aggregates that pass through sensors as oversized particles. This inflates large-diameter size classes while simultaneously underestimating true grain counts. Although surfactants and sonication are often used to mitigate aggregation, their effectiveness varies substantially among species, including several *Melastomataceae* that produce pollen with persistent adhesive properties (Vallejo-Marín, 2022).

Moreover, liquid particle counters share a fundamental limitation with most traditional methods in that they are destructive. Once pollen has been suspended, processed, and counted, the sample is effectively consumed, limiting opportunities for repeat measurement or independent verification without recollection. These limitations complicate large comparative studies and obscure real-time patterns of pollen release.

## A conceptual shift: counting pollen in the air

The central innovation of Lazarus *et al.* (2026) lies in repurposing a commercial handheld airborne particle counter (ParticlesPlus model 8506-30)—typically used for environmental particulate (contamination) monitoring—as a quantitative tool for pollen biology. The instrument draws air through a laser-based sensor, reporting size-resolved particle counts in real time. The authors exploit a simple but powerful insight: pollen released during floral sonication or wind disturbance behaves as an aerosol, analogous to other airborne particulates (Niklas and Spatz, 2019). This approach also provides an answer to a persistent issue in buzz pollination research—the directionality of pollen ejection. In *Solanum* anthers, exiting pollen jets are relatively predictable, but in many *Melastomataceae* the orientation and geometry of stamens cause pollen to be expelled in highly complex three-dimensional patterns (Lazarus *et al*., 2026). Traditional methods that rely on collecting ejected pollen in an Eppendorf tube—particularly in species where the anther pores are offset, rotated, or shunted by stamen curvature—can miss large fractions of the released pollen during mechanical excitation. This directional bias was visually confirmed by high-speed videography in Lazarus *et al*. (2026). Rather than collecting pollen into tubes, suspending it in liquid, and processing it post-hoc, the airborne approach samples pollen directly from the air at the moment of release. This eliminates nearly all intermediate steps: no tubes, no dilution, no sonication, and no centrifugation. The pollen cloud itself becomes the sample. In doing so, the method reframes pollen not as a static quantity to be recovered after dispersal, but as a dynamic flux that can be measured in real time using a handheld counter (Video 1).

This conceptual shift is particularly significant for buzz-pollinated systems. In such taxa, pollen release is driven by bee-generated vibrations and strongly influenced by anther geometry, pore placement, and stamen orientation (Russell *et al*., 2017; De Luca and Vallejo-Marín, 2019). Traditional collection methods, such as capturing ejected pollen in tubes or on slides, may miss substantial fractions of pollen when ejection vectors are misaligned with the collection apparatus. Lazarus *et al.* (2026) demonstrate, using high-speed videography, that pollen jets can follow complex three-dimensional trajectories that vary among species and even among individual flowers. By actively drawing air into the sensor, the handheld counter reduces sensitivity to ejection directionality.

## Accuracy, variance reduction, and avoidance of clumping artifacts

Across most species examined, Lazarus *et al.* (2026) report median pollen counts 25- to 35-fold higher than those obtained with liquid particle counters, accompanied by substantially reduced coefficients of variation. In many cases, airborne counts approximated normal distributions, whereas liquid-based counts were frequently bimodal or strongly skewed. Such distributional differences are consistent with stochastic pollen capture and aggregation effects associated with liquid suspension methods (Ruedenauer *et al*., 2016).

A key advantage of the airborne approach is that pollen is counted prior to hydration. By sampling pollen in the state in which it is naturally released, the method avoids many of the physicochemical processes that drive clumping in aqueous suspension. Lazarus *et al.* (2026) show that particles in the >30 m size class—interpreted as putative aggregates—are rare and only weakly correlated with total pollen counts across species (*r*^2^ < 0.15). This suggests that coincident or aggregated particles contribute minimally to total counts under their experimental conditions.

## Methodological caveats and practical considerations

Despite its advantages, airborne pollen counting is not without limitations. At extremely high pollen release rates, multiple grains may pass through the laser simultaneously and be registered as a single particle, potentially leading to underestimation. Although Lazarus *et al.* (2026) report that this effect is minor under their experimental conditions, its magnitude may increase in species with exceptionally large pollen loads or during intense vibrational excitation.

Background airborne particulates also warrant consideration. In controlled laboratory settings, dust and debris in the relevant size range are minimal, but in some field environments—such as agricultural landscapes or arid regions—baseline particle loads may require correction through background subtraction or size filtering. In addition, airborne sampling remains inherently ephemeral. Once the pollen cloud has passed through the sensor, no physical sample remains for repeat counting or archiving, complicating formal assessments of repeatability.

Cost represents another potential barrier. At approximately US$6000, the handheld airborne counter used by Lazarus *et al*. (2026) may be inaccessible to some laboratories, particularly those in low- and middle-income countries. Even in the USA and the EU, it is often difficult to obtain funding for instrumentation-only grants. Nevertheless, this cost remains substantially lower than that of many automated liquid particle counters, and the dramatic reduction in labor may offset initial investment over time. Hemacytometers remain the most cost-effective pollen counting aid (typically at US$85 to US$250 per unit) but are also somewhat laborious to use.

## Implications and future directions

By reframing pollen as an airborne signal rather than a liquid sample, Lazarus *et al.* (2026) introduce a methodological shift with broad implications for pollination biology, aerobiology, and plant reproductive ecology. Real-time quantification of pollen release opens up new avenues for studying pollen-dispensing schedules, interspecific differences in pollen ejection dynamics, and the functional consequences of floral morphology.

The approach is particularly well suited to buzz-pollinated systems, where pollen release is tightly coupled to vibration frequency, amplitude, and anther mechanics (De Luca and Vallejo-Marín, 2019; Vallejo-Marín, 2022). More broadly, airborne counting may facilitate integration of pollen biology with biomechanical and aerodynamic models of dispersal (Niklas and Spatz, 2019). As this method is further validated across taxa and environmental contexts, it has the potential to resolve longstanding questions about how plants regulate pollen release and how pollinators interact with floral structures to access pollen rewards.
